# Editorial: Marine Microorganisms and Their Enzymes With Biotechnological Application

**DOI:** 10.3389/fmicb.2022.901161

**Published:** 2022-04-18

**Authors:** Haijin Mou, Francesco Secundo, Xiaoke Hu, Benwei Zhu

**Affiliations:** ^1^College of Food Science and Engineering, Ocean University of China, Qingdao, China; ^2^Istituto di Scienze e Tecnologie Chimiche “Giulio Natta”, CNR, Milan, Italy; ^3^Yantai Institute of Coastal Zone Research, Chinese Academy of Sciences (CAS), Yantai, China; ^4^College of Food Science and Light Industry, Nanjing Tech University, Nanjing, China

**Keywords:** marine microorganism, enzymes, biotechnological application, heterologous expression, coding gene

The understanding and exploration of marine microbial resources is a new area with enormous potentialities in numerous fields of research and application. Due to the extreme and fluctuating salinity, pressure, and temperature of the marine environment, marine microorganisms are forced to develop unique metabolic pathways to adapt to this environment, producing enzymes that show different properties compared to the analogous ones obtained from terrestrial sites. Besides, being an important source of new active substances, and thanks to the disclosure of the particular the genome of marine microorganisms, as well as to the application of marine microbial engineering and enzyme engineering technologies, marine microorganisms and their enzymes show important development prospects in the agri-food industry, medicine, and other biotechnological fields. At present, the research on microbial physiology and metabolism under extreme marine conditions has made remarkable progress, and the application of this knowledge has also been applied for the restoration of the marine environment.

This Research Topic, which includes 11 original research articles and a review article, focuses on the discovery, properties, physiological characteristics, and industrial applications of new and valuable marine microbial enzymes.

Five articles focus on marine microbial hydrolases with potential application value in the preparation of functional oligosaccharides. Cao et al. described the properties of βκ-carrageenase for the degradation of hybrid carrageenan–furcellaran. Oligosaccharides from carrageenan—a group of important food carbohydrates with high sulfate content—have attracted extensive attention for their possible physiological functions. This work cloned and expressed a novel furcellaran-hydrolyzing enzyme from the marine bacterium *Wenyingzhuangia fucanilytica*. Considering the structural heterogeneity of carrageenan, this new enzyme was named as “βκ-carrageenase” instead of “furcellaranase.” Liang et al. designed a consensus sequence design strategy to improve the catalytic ability of a thermostable and acidophilic β-mannanase. Based on the amino acid sequence consistency analysis compared with the reported mannanase, the authors constructed several mutants that showed 303.0, 280.4, and 210.1% increased k_cat_/K_m_ and proved that this approach is an effective way to improve the performance of marine enzyme preparations. Ding et al. expressed an amylase from the marine bacterium *Saccharophagus degradans*. The recombinant enzyme can produce maltooligosaccharides with high specificity, providing a promising tool for the preparation of nutritional factors. It is worth noting that this enzyme also shows excellent cold adaptation and salt tolerance, a particularity typically observed for marine-derived enzymes. Zhou et al. characterized a new β-galactosidase from the marine bacterium *Bacillus* sp. BY02. The enzymatic properties show that the “Cys-Zn” motif plays an important role in the structural stability and catalytic function. Furthermore, Gal42 shows effective lactose hydrolysis activity, which makes it a potential candidate in food technology. Wang J. et al. characterized an efficient chitosanase from *Bacillus mojavensis*, which is suitable for the preparation of chitosan oligosaccharides. A high-efficiency recombinant chitosanase was obtained through homologous expression in *Pichia pastoris* X33. After 15 min of reaction, the total yield of chitosan oligosaccharides reached 92.3%, which was higher than the efficiency of most chitosanases previously reported.

In addition to enzymatic hydrolysis to produce marine characteristic carbohydrates, microbial fermentation is also considered to be a promising bioengineering technology. When extracting chitin from shrimp and crab shells, the first step is usually to remove protein and minerals. Although the widely used acid and alkali treatment method is effective, it has brought more serious environmental pollution problems. Therefore, many conventional technologies have turned to bioengineering technologies, such as the application of microbial fermentation. Xie et al. investigated the successive two-step fermentation to extract chitin from shrimp shells. Deproteination and demineralization can be effectively realized by the fermentation of a protease-producing strain, *Exiguobacterium profundum*, and a lactic acid-producing strain, *Lactobacillus acidophilus*. This work provides an environmentally friendly strategy for the industrial extraction of chitin.

Two articles focus on marine microbial lyases, including alginate lyase and hyaluronate lyases. Because many marine polysaccharides have a uronic acid scaffold, by lyases (common in marine microorganisms) it is possible to degrade them through a β-elimination mechanism, generating unsaturated disaccharides with a C_4_-C_5_ double bond at the non-reducing end. Wang X. et al. investigated two hyaluronate lyases from a marine bacterium *Vibrio alginolyticus* LWW-9 and proposed a model for the complete degradation of hyaluronic acid. Liu et al. expressed in *Yarrowia lipolytica* an alkaline alginate lyase that performs unique heat recovery. The enzyme mainly produces DP1–DP2 oligosaccharides, making it a potential candidate enzyme for monosaccharide and disaccharide industrial production.

One article focuses on the heterologous expression of cyclodextrin glycosyltransferase and its application in the transformation of 2-O-α-D-glucopyranosyl-L-ascorbic acid (AA-2G). Song et al. reached the maximal AA-2G yield of 28 g/L, which is particularly interesting for the industrial production of AA-2G.

Three articles are about marine ecological microorganisms and environmental bioremediation. Meyer-Cifuentes and Öztürk reported a marine mesophilic MHETase-like enzyme, which can hydrolyze polyethylene terephthalate and its intermediate degradation product, mono-2-hydroxyethyl terephthalate, to monomers. This study is of certain significance to eliminate the threat of plastics in the marine environment. In addition, Wang Y. et al. reported a mini-review to introduce the research advances in 2-haloacid dehalogenases. Changfei et al. found that the restoration of *Suaeda salsa* in coastal wetland increased bacterial taxa with the ability to degrade symbionts and aromatic compounds, which was helpful to improve the bioremediation role.

The research results collected on this Research Topic are not exhaustive. At present, marine microorganisms are considered as a novel source of new enzymes because of their genetic and biochemical diversity. Marine microorganisms produce physiological mechanisms to adapt to marine extreme environments such as cold and/or salt tolerance, surviving in harsh conditions. Compared with terrestrial homologs, marine microbial enzymes have unique structural characteristics, which show high flexibility, solubility and substrate binding ability. With the help of whole genome technology, gene recombination, rational design and synthetic microbiology, the research of marine microorganisms and enzymes is developing rapidly. A variety of enzymes with peculiar properties have been isolated from marine bacteria, actinomycetes, fungi and even bacteriophages, some of which have achieved heterologous expression and industrial application. Marine microorganisms and their enzymes will be expected to generate great industrial value, with an important impact on human life in the future.

## Author Contributions

All authors listed have made a substantial, direct, and intellectual contribution to the work and approved it for publication.

## Conflict of Interest

The authors declare that the research was conducted in the absence of any commercial or financial relationships that could be construed as a potential conflict of interest.

## Publisher's Note

All claims expressed in this article are solely those of the authors and do not necessarily represent those of their affiliated organizations, or those of the publisher, the editors and the reviewers. Any product that may be evaluated in this article, or claim that may be made by its manufacturer, is not guaranteed or endorsed by the publisher.

